# Synthetic Mutualism and the Intervention Dilemma

**DOI:** 10.3390/life9010015

**Published:** 2019-01-28

**Authors:** Jai A. Denton, Chaitanya S. Gokhale

**Affiliations:** 1 Genomics and Regulatory Systems Unit, Okinawa Institute of Science and Technology, Onna-son 904-0412, Japan; jai.denton@oist.jp; 2 Research Group for Theoretical models of Eco-Evolutionary Dynamics, Max Planck Institute for Evolutionary Biology, 24304 Plön, Germany

**Keywords:** cross-feeding, mutualism, intervention strategies, ecosystem engineering

## Abstract

Ecosystems are complex networks of interacting individuals co-evolving with their environment. As such, changes to an interaction can influence the whole ecosystem. However, to predict the outcome of these changes, considerable understanding of processes driving the system is required. Synthetic biology provides powerful tools to aid this understanding, but these developments also allow us to change specific interactions. Of particular interest is the ecological importance of mutualism, a subset of cooperative interactions. Mutualism occurs when individuals of different species provide a reciprocal fitness benefit. We review available experimental techniques of synthetic biology focused on engineered synthetic mutualistic systems. Components of these systems have defined interactions that can be altered to model naturally occurring relationships. Integrations between experimental systems and theoretical models, each informing the use or development of the other, allow predictions to be made about the nature of complex relationships. The predictions range from stability of microbial communities in extreme environments to the collapse of ecosystems due to dangerous levels of human intervention. With such caveats, we evaluate the promise of synthetic biology from the perspective of ethics and laws regarding biological alterations, whether on Earth or beyond. Just because we are able to change something, should we?

## 1. Introduction

Synthetic biology can be considered the development of biological systems that behave predictably via introduction of well-characterised genetic modifications [1,2]. As the nexus of several disciplines, the definition of synthetic biology is subject to considerable revision reflecting varying perspectives. Often cited for industrial or health applications, synthetic biology is also a powerful tool for studying fundamental biological processes. It includes the study of complex population dynamics and ecological interactions, like mutualism, via construction of biological systems with predictable behaviours.

Mutualism occurs when two species cooperate to their reciprocal benefit. This is a subset of symbioses, which embrace all types of interactions between different organisms. Mutualistic interactions are fundamental to the origin and evolution of life, seen across all scales of organisation (from molecules to complete ecosystems) [3,4]. For example, molecules can replicate themselves, as well as other molecules [5]. Cells can provide resources to one another, sometimes even at considerable metabolic cost [6]. Moreover, the diversity of mutualistic interactions, such as ant-plant, coral-dinoflagellate, seagrass-bivalve, leguminous plant-rhizobia, squid-vibrio and deep sea mussel-chemosynthetic symbionts [7,8,9], highlight the importance of symbiotic relationships in nature. Even extreme environments are rarely home to single species, but rather to consortia [10,11,12]. Initially, simple mutualistic relationships can evolve into these consortia comprising a range of biological interactions, from competitive to cooperative, forming complex symbiotic networks [13].

Working together, mutualists can thrive in environments that would be inhospitable to individual species. Deep sea mussels and their chemosynthetic symbionts manifest the power of mutualism in extreme conditions [9]. Chemosynthetic bacteria, feeding on reduced compounds seeping from deep sea vents, fix carbon into organic matter, providing the mussels with a food source. The symbiosis between the mussels and sulphur- and methane-oxidising bacteria has helped them colonise hydrothermal vents and cold seeps, overcoming severe environmental pressures.

Environmental pressures can also promote changes in symbiotic interactions—from passive by-product reactions to mutualisms [14]. Structure and change in environments could thus be intimately related and perhaps causal to major transitions of life on earth such as symbiosis/endosymbiosis [15] or even the origin of life [16]. Seismic shifts in the environment, such as the Great Oxidation Event could alter the trajectory of life, leading to survival and evolution of organisms that can work together. The rise of oxygen effectively poisoned the anaerobic organisms. One way or surviving the apocalypse was to associate with other organisms who could survive in the presence of oxygen. Such a process could lead to the origin of major transitions in life such as multicellularity [17]. In essence, mutualism is crucial in shaping and maintaining the diversity of life that we see around us, but these interactions are now at risk.

The diversity of life is facing an ecological collapse due to anthropogenic activities. Mutualisms, especially long-lived ones, are under constant ecological threat. Changing environmental conditions can drive stable mutualisms to devolve into parasitism [18]. Rampant use of pesticides has affected symbioses between plants and rhizobia, which have evolved over millions of years [19]. Eutrophication of oceans is destroying the mutualism between dinoflagellates and corals, essential for the maintenance of not just corals, but the entire ecosystems they support [18,20,21]. While a high degree of anthropogenic disturbance is responsible for such destruction, guided intervention techniques could potentially be used to intervene and stabilise some of these collapsing relationships [22,23]. As an example, it could be possible to modify dinoflagellates that function as coral symbionts, to withstand higher temperatures [24]. Moreover, synthetic biology could help us address some fundamental questions about mutualistic interactions. Specifically, how do they arise in the first place? What keeps them from degenerating, and how can mutualism become parasitism?

In this manuscript, we review currently available synthetic biology experimental models and their theoretical analogues. Taken together, the models afford us a glimpse into the immensely diverse future use of synthetic biology. We highlight studies leading the field in using synthetic biology to explore the fundamental organising principles of mutualistic relationships. Developments in experimental systems integrated with theoretical studies are reviewed. As an application, the use of synthetic symbiotic systems to stabilise fragile ecosystems is discussed. Beside science, we venture a cautious opinion about legal and ethical implications of such applications. Although we are now able to implement ecosystem-level change, should we?

## 2. Experimental Modelling

There are numerous systems used to study mutualism, each illuminating specific aspects of this complex interaction. Studies of mutualism can employ either a top-down approach, studying an entire complex network and then dissecting it, or a bottom up methodology, examining a single interaction and building on that knowledge. Being embedded in ecological niches, naturally occurring systems are intricately connected with interactions of which we may be unaware. While enabling us to understand dynamics of complete ecosystems, the effects of individual interactions can be discounted. In contrast, tractable synthetic laboratory-based systems allow dissection of individual interactions without the confounding effects of entire networks. There are also numerous degrees of complexity between these two extremes, including naturally occurring systems in a laboratory, subsets of naturally occurring systems, or experimental evolution of novel systems.

### 2.1. Natural, Selected or Evolved Systems

Numerous natural systems exist for the study of mutualism. Attractive examples abound, such as humans and honeycatchers, plants and rhizobia, and corals and dinoflagellates, to name just a few [7,25,26]. Even from deep-sea hydrothermal vents [10,27,28], studies have been extremely fruitful in deciphering interactions between resident life forms [29], most of which are fueled by symbioses with chemoautotrophs. Microbial analysis on recently settled lava-flows demonstrates the power of microbial consortia in pioneering harsh environments [30,31]. Specifically, colonization of these lava flows by bacteria that oxidise inorganic compounds, like ${\mathrm{SO}}_{4}$, fixes compounds, like $N_{2}$ and ${\mathrm{CO}}_{2}$, required for other heterotrophic organisms [30,31,32]. While living systems can be analysed under laboratory conditions, some of the oldest fossils available, stromatolites, unsurprisingly were also consortia of microbes [33].

**Figure 1:**
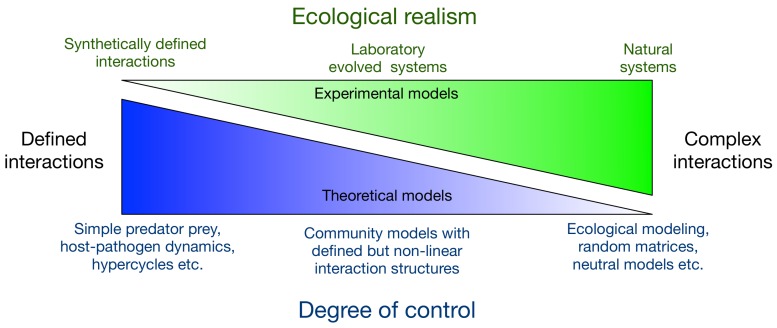
The trade-off between ecological realism and the degree of control over the interspecies interactions afforded by synthetic biology.

However, not many of these systems lend themselves to laboratory investigations. Because such systems are often studied in native or semi-natural conditions, they are usually complicated by confounding factors. Specifically, cooperation is often not limited to merely two interacting species, as several species can interact simultaneously [34]. This situation is perhaps best exemplified by microbiomes. Microbiomes, the collection of microbes within a host, fulfil crucial roles in the health of the host and in principle can help to modulate numerous biological functions [35]. Artificial selection through co-culturing and selection of interacting components has proven effective in developing experimental bacterial systems [3,36,37]. These systems and others *derived* from experimental evolution are sometimes called synthetic systems. In this manuscript, we define synthetic systems as those that rely on engineered genetic modifications (Figure 1).

### 2.2. Synthetic Systems

The predictability and tuneability of synthetic biology allow for a precise studies of complex biological processes [38,39,40]. Synthetic biology can minimise effects of stochastic factors, giving greater control for studying fundamental biological questions. This control extends to the study of mutualism, as various systems in both yeast and bacteria have been developed. These synthetic mutualistic systems usually rely on strains unable to synthesise specific nutrients (known as auxotrophs), but that over-produce different nutrients to levels that allow cross-feeding [41,42,43,44]. When grown together, these strains require a nutrient produced by a partner strain. Synthetic mutualism can also rely on strains that influence the survival of another strain via secretion of non-nutrient compounds. For example, complex pathways conferring antibiotic resistance can be distributed between two strains, which must be co-cultured for resistance [45]. For simplicity, these systems tend to be of the same species, but they do not need to even belong to the same biological kingdom. These systems can be grown in highly variable conditions to explore interactions. This permits the system user to change component ratios quickly, media composition by the addition of a required nutrient or even, when grown on solid media, spatial constraints of the strains [41,42,43,44,45]. The defining characteristic of these synthetic systems, such as those listed in Table 1, is that the interactions between components are completely artificially determined. If we classify the experimental systems to be far from naturally occurring communities, then systems with synthetically engineered interactions lie at the far end of the spectrum (Figure 1).

Several synthetic bacterial cross-feeding systems have been established. Due to its thorough characterisation, the model organism, *Escherichia coli*, is a common starting point for the development of these synthetic systems. Hosoda et al. [43] developed one of the first mutualistic systems. In this two-strain system, one strain was auxotrophic for leucine, but overproduced isoleucine, whereas the opposite was true of the second strain. These strains were unable to grow without supplementation of the media or the presence of the other strain. Pande et al. [44] established a bacterial cross-feeding system with *Acinetobacter baylyi* and *E. coli* strains that were capable of mutualism. Both species were engineered with tryptophan or histidine auxotrophy and the ability to cross-feed the complementary nutrient. This setup provides an interspecific model of mutualism.

Nearly identical systems that rely on auxotrophy and overproduction have been developed in the eukaryote—*Saccharomyces cerevisiae* (yeast). Due to a well-characterised sexual reproductive cycle, an abundance of tools for manipulation, and similarities to other eukaryotic cells, yeast is commonly used in synthetic biology. With short generation times, highly tractable yeast model systems can be rapidly developed and combined. Two yeast-based cross-feeding mutualistic systems have been published, each having two cooperating components [41,42]. Like those described above, these systems both rely on cooperating auxotrophs and overproduction. In unsupplemented media, both strains can grow only as a pair. These strains contain a type of mutation known as feedback resistance. Biosynthetic pathways tend to be repressed by the final metabolite within the cell, but these mutations confer immunity to this feedback mechanism, and result in an abundance of a particular pathway product [46,47].

Using synthetic biology, the behaviour of interactors can be precisely controlled. This precision is similar to how theoretical models are often constructed, with the role of entities or interactions between them predetermined. An example of this are simple predator-prey models. There are only two interacting species, a predator and prey. The prey is eaten by the predator which determines the population dynamics of the system along with the prey growth rate and the predator death rate. This simple model is far from reality [48]; however, it is an indispensable model of theoretical ecology, the Lotka-Volterra dynamics. Experimental systems are being constructed with almost the same amount of precision as is typically assumed in the theoretical models (Figure 1). Going forward an integrated modelling approach is possible where both the experimental and theoretical models are close analogues of each other. We can thus build theoretical models to test assumptions of the interaction structure and experimental models to test predictions of theoretical assumptions.

## 3. Integrated Modelling

Theoretical modelling of community ecology ranges from specific interactions between the involved species, such as the host-pathogen, predator-prey models [48,55,56] to random interactions able to explain species co-existence via a neutral models [57,58,59]. While the general models lack predictive power due to their inherent complexity, this is typically closer to the behaviour of natural systems. Synthetic symbiotic systems, on the other hand, have predefined interactions between mutualists. While such precise interactions are now a reality, typically they have been integral assumptions of most theoretical models Figure 1 (left edge). The level of detail, e.g., individuals, ecology or environmental variation, at which the mathematical models are developed can vary greatly [60].

Considering extreme ecology, where except for the two concerned species no others exist, we can use mathematical models developed for such straightforward cases. Proposed by Manfred Eigen [5] and then developed together with Peter Schuster [61], the hypercycle is a system of replicators, the members of which can auto-catalyse as well as cross-catalyse. A hypercycle is a closed network of reactants in which each reactant type is capable of autocatalysis. Furthermore, replication of each reactant can be catalysed by other reactants as well. Hypercycles have been implicated at the origin of life as well as in forming complex communities [62,63]. The theory of hypercycles can be further connected to evolutionary game theory, where many results from one field can be translated into the other [64,65], for example, the conditions for the stability of such connected systems.

Hypercycles thus provide us with a general model of two (or more) species locked in symbiotic relationships. Theoretical developments in the field have developed variations on this model [66,67,68,69,70], but it was not until recently that integrated modelling of systems akin to hypercycles was possible. Experimental evidence on spontaneous network formation by RNA provides firm support of hypercycle stability [71,72].

While theoretical studies abound [73,74], simple cross-feeding networks together with theoretical frameworks have the potential to test the predictions of the theory and the underlying assumptions of the models as well [41,42,49,51] (Table 1).

### 3.1. Bridging the Gap

As an example of how synthetic biology and theoretical biology inform each other, we take the example of the model of a simple hypercycle as mentioned above. The hypercycle is an extremely abstract conceptual model, with reactants replicating themselves and facilitating replication of another. The Hosoda et al. [43] system of leucine and isoleucine sharing between the engineered *E. coli* strains works in a similar fashion. Amor et al. [49] used the strains precisely in the form of a hypercycle (Figure 1). Firstly, hypercycles assume that the reactants only replicate themselves and help the replication of another reactant to replicate, with no other interspecific consequence. The engineered *E. coli* strains satisfy this assumption owing to their synthetic nature. Secondly, the hypercycles are predicted to be stable against parasites when a spatial structure is present. Amor et al. [49] confirmed this prediction experimentally using the same engineered strains.

Experimental systems can be used to explore plasticity of interactions via environmental manipulation. The yeast systems mentioned above are clear examples [41,42]. The Müller et al. [42] system, originally used to explore the effects of genetic drift on mutualists undergoing spatially expansive growth on solid media, was used to explore how nutrient availability shapes mutualism [14]. Therein a simple phenomenological model, using the growth rates from the experimental system, showed that under varying levels of supplementation, the nature of the interaction between the system components changed [14]. This was subsequently confirmed with the experimental system. System results lay on the spectrum from an ecological collapse to competitive exclusion via mutualism and parasitism depending on the supplementation levels. A similar, integrated assay in the system of Shou et al. [41] explored the impact of environmental disturbances (supplementation levels) [51]. Such integration of theory and experiments based on synthetic systems provides insights into real ecosystems and could guide the design of new synthetic systems.

### 3.2. Towards More Complex Integration

Although simple systems provide a start, increasing the number of interactors in a mutualistic network, given the cross-feeding reactions possible [75], is a theoretically challenging task [76,77]. For two interacting species, it might be possible to use a simple pairwise modelling approach, in which the cumulative effect of one species on the other is parameterised. This technique has been hugely successful in predicting dynamics of communities in which individual pairwise interactions are known [76]. The technique has clear advantages over classical ecological models based on generalised Lotka-Volterra dynamics, which require a massive amount of data to fit a large number of parameters. Moreover, problems of classical models are further magnified because they often cannot capture the dynamics of pairwise interactions [77]. Thus models need to include flux balance analysis, in which not just the details of interacting species, but those of the intermediate metabolites are also taken into account [60,78,79,80].

### 3.3. Including the Complete Ecology

Ecology, as traditionally considered in evolutionary models, is typically limited to population dynamics [81]. For example, although Lotka-Volterra models are considered ecological, they consider only interacting organisms. However, the ecology of an organism embraces abiotic factors as well [82]. Thus inclusion of the whole (biotic as well as abiotic factors of the) ecology is necessary in order to integrate theory and experimental models.

Beside the mutual support of two interactors via production of mutually beneficial metabolites, the system also requires an ecological niche. If population densities of mutualistic partners are inadequate, mutualism is hard to sustain [16,83]. This problem highlights the necessity of a physical structure akin to a “warm little pond” for concentrating primary mutualists [84]. Without physical structure, such as a vesicle or a membrane, to contain mutualists, or a physical substrate providing a common ecological niche, it would be hard for the population to aggregate sufficiently to kick-start necessary mutualistic reactions [85,86]. This underscores the importance of conducive ecology, a spatial structure offering just the right amount of flux, enough to concentrate hypercycle components so as to initiate amplification of the cycle [49]. In synthetically generated mutualism, compartmentalisation and its effect on sustainability and performance of RNA hypercycles have also been experimentally tested [72,87].

### 3.4. Extension to Evolved Symbiotic Systems

With their highly controlled genetics, experiments with synthetic mutualism systems are rarely conducted over time frames that permit selection to act (outside of the experimental design). However, systems that lack wholly defined interactions, such as selected, evolved or natural systems (see above), can evolve a well-known phenotype, “cheats”. A common challenge for a mutualistic community is exploitation by a cheater strain that benefits from mutualistic interaction but fails to contribute. The problem of cheats in a hypercycle is well understood when we think of mutualisms [63]. Phenomenological means of avoiding cheats are via group selection, kin selection, spatial segregation and direct and indirect reciprocity [88]. Mechanistically however these solutions are not always possible (e.g., the concept of “reciprocity” does not exist for molecules). Thus mechanistic arguments (such as spatial segregation, diversity in metabolite uptake rates and kin recognition mechanisms) are proposed as appropriate for the system [89]. Concerning cross-feeding networks, if we compartmentalise mutualists, then it might be possible to improve on their cooperativity—a phenomenological solution. Mechanistically this can work by privatising the public good and streamlining interactions leading to strengthened cooperation [44,90]. Connecting the RNA experiments mentioned above, long-term evolution can result in more efficient RNA cooperation, even when seemingly parasitic RNA elements are present. Therefore, compartmentalisation or spatial segregation can promote transition from one level of organisation to another. For the first reactions necessary to start life, hydrothermal vents may have provided such porous, spatially segregated compartments [16], where segregation might not have been just spatial, but thermal as well. Synthetic biology can be used to generate spatial structures necessary to promote synthetic mutualism [91,92,93,94].

We have reviewed the theory of mutualistic interactions between species starting with elementary models. Presently in biology, immense amounts information can parameterise complex modelling techniques, such as metabolic network theory [80,95,96]. We choose to focus on simple models involved in testing assumptions of general ecological concepts. Even considering different modes of theoretical development, it is clear that both theoretical and experimental developments have reached the point where we can now not only understand underlying principles of mutualism, but can even guide and direct it. Simple mathematical models can now be experimentally tested using synthetic biology in the lab [42,49,51,97]. With this information, we can more closely model naturally occurring mutualisms. Without understanding long-term consequences of anthropogenic activities, mutualistic systems have been adversely impacted by cascading ecosystem effects. Perhaps with increased knowledge from integrative experimental and theoretical models, we can propose remediation to stave off ecological collapses [51,98].

## 4. Future

As we continue to innovate and integrate frameworks to study biological processes, the questions we tackle grow in scale. The advent of synthetic biology is shaping the complexity of our models and our ability to generalise their use. With time, we hope that these models will allow accurate predictions of ecosystem-scale events. When this does happen, we will face the prospect of intervening to change or at least slow these events, but should we?

### 4.1. Better Systems

Although theoretical biologists have long enjoyed considerable freedom in the interactions they model, experimental systems to support these models have eluded researchers. However, as stated above, synthetic biology is allowing development of experimental systems with levels of control previously only seen in theoretical biology [99,100] (Figure 1). As the techniques and technologies continue to develop, so does this control. We are on the verge of developing large complex synthetic networks of interacting microbes.

Further control of models of mutualism will arise via the considerable collection of tools at our disposal. Although the systems described above are powerful, researchers are still limited in the exact dynamics explored. For example, although it is possible to modulate the cost-benefit ratio of a public good by environmental supplementation, finely regulated genetic control will allow more complex dynamics. Examples include promoters that are inducible via the addition of compounds like tetracycline, niacin, or miRNAs, or through processes that alter protein activity, like temperature-sensitive inteins [93,101,102,103]. In each of these cases, if genes linked to mutualism, like those responsible for cross-feeding, are under the control of these systems, population dynamics can be altered via environmental changes. There is also a growing body of genetic tools for establishing spatial factors within growing populations [91,92,93,94]. Moreover, expression systems are continually being refined to improve their predictability by adjusting the regulatory structure, for example, by modulating ribosome binding sites, introducing feedback, or influencing chromatin structure [104,105,106]. This will afford researchers with an unparalleled level of experimental control, and facilitate the precise shaping of mutualism during laboratory-based experiments.

At the forefront of these new systems is work in which several 2-way relationships were developed [45]. Kong et al. [45] developed six relationships, commensalism, amensalism, neutralism, cooperation, competition, and predation, and predicted behavioural dynamics. They went on to develop several more in silico consortia that built on these relationships. Synthetic biology emphasises modular and predictable behaviour that can be applied equally to modules of ecosystems [60]. A modular design of synthetic systems will provide a way to rapidly develop novel microbial consortium-based systems.

Although synthetic systems are often grown on solid media to explore spatial dynamics, development of synthetic biofilms or cell adhesion dynamics potentially permits exploration of these dynamics in liquid culture. Finally, the most significant advance in these model systems will likely be the addition of additional system members. Natural mutualistic interactions, especially in microbiome research, can involve a considerable number of interacting partners. Non-linear effects of multiple partners are typically not captured with theories developed for two-strains [77,107]. Knowledge from theoretical models of the importance of ecological factors (biotic and abiotic) affecting the stability of a community, together with experimental data, can help us design better systems that work for us rather than against us. Consortia of microbes corroding pipelines of oil reservoir production systems illustrate this point. Differences in temperature select for a different microbial composition, impacting biocorrosion of the pipes [108].

### 4.2. Intervention

As knowledge of a particular system increases, so does ease in predicting the effect of an intervention. A well-targeted intervention can have a considerable impact on an ecosystem. This impact can be so substantial that it influences multiple trophic levels (a trophic cascade). Historically speaking, when discussing intervention in biological systems, we usually refer to manipulating an ecosystem for an economic (e.g., agricultural) benefit. There has been considerable success with the control of species detrimental to agricultural interests like screwfly [109], prickly pear [110], or fruit fly [111]. Similar approaches, albeit on a smaller scale, have also been used for conservation. Removal of an invasive animal from a location like an island, where it cannot easily repopulate, allows restoration of the natural ecosystem.

Similarly, the reintroduction of an animal can have a considerable positive impact on ecosystem restoration. The most prominent example of this is the reintroduction of the grey wolf to Yellowstone National Park, which resulted in a top-down trophic cascade [112]. It is important to note, however, that the effects of this reintroduction, while arguably beneficial for overall ecology and surrounding agriculture, were not fully anticipated and may not be for decades [113]. Although an intervention success story, this does serve as a clear warning that some outcomes can be nearly impossible to predict. There has been a growing discussion about the merits and dangers of GMO technology in conservation [23,114,115], a topic that will need increasing discussion.

#### 4.2.1. Modifying Life

Technology facilitating modification of life is now over 40 years old. From the first transgenic modification of a virus in 1972, there was only a two-year gap until the first genetically modified mouse [116]. The first genetically modified plant, tobacco, followed in 1983, 11 years after the first GMO [117]. These technologies generally involve the insertion of foreign DNA into the target organism or the targeted deletion/mutagenesis of a specific gene. Technology has continued to mature, but since the discovery and development of CRISPR techniques, the modification process has become much faster, more precise, and applicable to a much broader group of species [118,119].

In principle, gene editing processes could be used to alter ecologically significant organisms, influencing their mutualistic interactions. However, stabilising a mutualistic interaction via genetic modification would require transforming an entire population of one or more mutualistic partners. Such an undertaking would require gene-drive or similar techniques, whereby the standard rules of inheritance are manipulated to ensure that a target gene (or system) spreads through an entire population [120]. There are several technologies for achieving this, but the most prominent is CRISPR-based mutation chain reaction [121]. This technology relies on CRISPR to copy a locus, containing the CRISPR construct and other payload genes, to the homologous locus. This technology can theoretically spread quickly through a population from a single individual. An alternative gene-drive technology is single locus underdominance [122]. This relies on the weakness of individuals heterozygous for the underdominant locus to drive the locus through the population. The exact release numbers required to guarantee fixation differ with the implementation in question, but are generally higher than the total number of individuals in a target population. Although more costly, underdominance is considerably easier to contain and potentially reverse [122,123,124]. These technologies are continually improving, but only a few studies explore their stability and long-term effectiveness [125]. Currently, none of these GMO gene-drive systems has been deployed in the environment.

The very aim of these technologies is the permanent alteration of a target population. Permanence is potentially required in any plan to intervene in stabilising a target mutualistic interaction. As such, any intervention would need (i) to have a clear technology in place to alter the target organism, (ii) an understanding of how any alterations would influence the target mutualism, and finally, (iii) a firm grasp of the law and ethical implications involved in changing a species.

#### 4.2.2. Law and Implications

Intervening to preserve or reintroduce a mutualistic interaction has the potential to reshape an ecosystem completely. Therefore, before attempting any intervention, a great many questions need to be answered. Not least of all is, “Is reversal possible?” While permanence might be desired for successful intervention, the idea of permanence may shift the balance away from the decision to intervene. However, this balance is significantly altered by interventions based on genetic modifications. Given the difficulty of accurately predicting interactions on the same trophic level, let alone those above or below, any decision needs assessment based on many criteria, considering the positions of all stakeholders carefully. The gravity of decision-making is greatly magnified when a genetic modification is involved and even further when gene-drive is used [115].

Should genetic modification be used to stabilise or even reintroduce a mutualistic interaction? Is this conservation? Forty years after the first genetically modified organism, considerable, albeit varying, controversy remains associated with their use [126]. Reflecting this, regulatory regimes guiding the use and deployment of GMOs vary from country to country. Perhaps the most familiar use of this technology thus far has been the development of novel varieties of agricultural crops. Although numerous animals have been modified, these are predominantly for research purposes and are far from use in the wild. The two most prominent released GM animals are the AquaBounty salmon [127] and the RIDL mosquito [128,129]. Compared to these GMOs, synthetically engineered ecosystems likely would have a considerably greater impact.

Current regulatory frameworks tend to focus on GMOs that are not expected to be permanent fixtures in the environment, or that are localised. GMO crops or specialised facilities, as in microbial production of insulin, are such examples. However, most jurisdictions are continually updating their regulatory frameworks to reflect both these realities and the changing technological landscape. Permanently altering the environment might be more acceptable if the environment in question was not one of concern. Thus an isolated extreme environment might provide a potential test for this approach.

#### 4.2.3. Altering the Extreme?

The ecology of our planet is being subjected to a virtual tsunami of catastrophes, most of which are anthropogenic. Escaping the natural speed of evolution via a cognitive revolution, humans have raced to the top of the food chain without allowing the ecosystem time to catch up [130,131]. To facilitate this catch-up, could even more extreme measures than ecosystem stabilisation be needed? We leap beyond altering a particular life form in a contained ecosystem. Should planetary-wide modification—terraforming—be considered? The need to focus on such a blue-sky concept is not a romanticised construct, but is becoming a need of our times [132].

Perhaps synthetic biology can help us restore drastic detrimental changes that we have imposed on a planetary scale. Intervention would be required on an enormous scale to deploy microbial communities needed to clean up pollution or restore lost symbiotic relationships. Alternatively, restoration could be achieved by endogenising a mutualistic function from one partner into the other partner, e.g., engineering nitrogen-fixing capability in plants without need for their mutualistic partners [133]. Developing novel organelles could be a concept that is potentially in the realm of synthetic biology [38]. However, perhaps we do not need to think of higher level transformations. If we have learnt anything from evolution, it might be that just as there have been major transitions in life, perhaps there could be similar evolutionary transitions in synthetic systems. In the spirit of [98], we consider terraforming first in the context of this planet before we look to space.

## 5. Conclusions

We have highlighted only a small fraction of the exploratory power that synthetic biology offers in seeking to understand complex population dynamics. Engineering and design principles being employed are developing modular systems that can be easily used, reused, and modified. This modularity, in turn, affords a level of control, when using these systems, that can keep pace with theoretical models. Theoretical models often assume predefined interactions between interacting components that can now be precisely engineered in synthetic communities. The closing gap between experimental and theoretical models allows us to test not just assumptions, but predictions of theoretical models. For example, if theory predicts that specific classes of interactions are necessary to stabilize a community under ecological perturbations, then we can design synthetic communities to test our understanding. This will help us better approximate workings of natural or evolved systems in the laboratory and perhaps eventually in the wild.

Current synthetic mutualistic systems have small numbers of interactors, usually two with additional strains acting in a bystander or cheating capacity. These systems are on the verge of being superseded. The most exciting developments in these synthetic mutualism systems will be the creation of more complex synthetic networks that will allow a higher degree of complexity to be modelled. Synthetic biology also brings the promise of incorporating different attributes in microbes. This perhaps includes the ability to digest novel substrates or to withstand radiation and other extreme conditions. Within earth’s most extreme conditions, we find consortia of microbes. This includes the low oxygenation of ponds covered with algae, extreme drought in the Atacama desert, or even in caves representing Mars-like conditions [12,30,31,134,135,136]. Learning from these natural systems, we could benefit from allocating super-traits among diverse taxa rather than building one super-organism. From an academic perspective, synthetic mutualism may also provide sets of bio-signatures to aid the search for extraterrestrial life [137]. Moreover, from an applied perspective it may be possible to construct cooperating systems that can survive not just extreme environments, but to provide a foothold for terraforming other worlds [138].

As we develop greater understanding of the complex dynamics underpinning whole ecosystems, it may be possible to move beyond mere observation. Unfortunately, interventions have not always gone according to plan [139]. Humans have intervened in natural processes for millennia. However, synthetic biology provides us with unprecedented power to edit the very fabric of the interconnectedness of life on earth. It is nearly impossible to predict all the outcomes of an intervention, but beside that, an even more critical question looms. Just because we have the technology to implement an intervention, should we? Here we are encouraging a dialogue regarding ecosystem intervention to restore mutualistic interactions. With better tools and a holistic understanding of systems of interest, it is necessary for interventions to be justified. We are unaware of the downstream effects of introducing synthetic constructs into the environment. As synthetic communities interact with evolving microbes, interactions are bound to change. If interactions become sophisticated enough then we might lose predictive power [107,140,141,142].

Development of an ecological equivalent of BioBricks has been suggested [143], in which ecosystems are simplified and components are small ecological motifs with clearly defined inputs and outputs [60,144]. The work by Kong et al. supports this modular view in their development of complex dynamics in silico from their established parts [45,93]. To overcome a loss of predictive power, this idea could potentially be extended. *Eco-blocks* could be developed that rely on complex mutualistic interactions, but have clearly defined inputs and outputs, e.g., a self-limiting eco-block that raises local environmental pH by oxidising ${\mathrm{SO}}_{4}$ in extreme environments while fixing nitrogen. Such an eco-block would allow colonisation of an extreme environment by organisms, like plants, that are capable of sustaining other organisms. Such a process could further facilitate localised bioremediation or even large-scale terraforming. Although it still does not escape the difficulty of predicting outcomes resulting from interactions with existing microbes, such systems could potentially be deployed where there is little or no interference [145].

Mutualistic relationships have the power to produce, sustain, and develop microbial communities. While mutualism might not seem to be a dominant interaction in many complex communities, it is essential at the inception. We have reviewed emerging synthetic mutualism models of microbes that have developed rapidly over the past decade. Experimental systems are now in sync with theoretical models, which have been employed in making predictions for decades. With synthetic biology, we are now in a powerful and fast hypothesis generation-testing loop. While already useful in the industry, the temptation of deploying this technology in the wild is immense. However, we provide a cautionary outlook through the lens of ethics and laws; necessary to make sure that we do not leap before looking.

## Figures and Tables

**Table 1:** Synthetic mutualism systems—experimental and theoretical. This table contains only those systems that rely on synthetic expression or gene modifications to establish mutualism. These studies are often accompanied by theoretical models. While most have a model designed to fit that particular system, some studies use experimental systems to test an existing theory e.g., Amor et al. [49].

Species	Theoretical Analysis	Citation
*S. cerevisiae*	Minimal birth death growth model	Shou et al. [41]
	Spatial frequency-dependent selection model	Müller et al. [42]
	including drift and explicit nutrient dynamics	
	Diffusion dynamics and individual-based simulation	Momeni et al. [50] based on Shou et al. [41]
	and explicit nutrient dynamics and environment	
	ODE and hybrid dynamical systems	Denton and Gokhale [51] based on Shou et al. [41]
	ODE and stability analysis	Hoek et al. [14] based on Müller et al. [42]
*E. coli*	Monod type growth kinetics based model	Hosoda et al. [43]
	ODE-based model with Monod kinetics	Kerner et al. [52]
	ODE-based population dynamics model	Amor et al. [49] based on Hosoda et al. [43]
	akin to hypercycles [53] but including diffusion dynamics	
*E. coli—A. baylyi*	No theory	Santala et al. [54]
	Individual-based model	Pande et al. [44]
*E. coli—D. discoideum*	ODE-based population dynamics model	Kubo et al. [36]
*L. lactis*	ODE-based population dynamics with explicit	Kong et al. [45]
	signaling molecules and nutrient dynamics and varied	
	ecological interactions	
